# Distribution of magnetic remanence carriers in the human brain

**DOI:** 10.1038/s41598-018-29766-z

**Published:** 2018-07-27

**Authors:** Stuart A. Gilder, Michael Wack, Leon Kaub, Sophie C. Roud, Nikolai Petersen, Helmut Heinsen, Peter Hillenbrand, Stefan Milz, Christoph Schmitz

**Affiliations:** 1 Department of Earth and Environmental Sciences, Ludwig-Maximilians University of Munich, Theresienstrasse 41, Munich, 80333 Germany; 2 Department of Psychiatry, Psychosomatics and Psychotherapy, Center of Mental Health, University Hospital Würzburg, Würzburg, 97080 Germany; 3 Ageing Brain Study Group, Department of Pathology, LIM 22, University of São Paulo Medical School, São Paulo, Brazil; 4 Department of Neuroanatomy, Ludwig-Maximilians University of Munich, Pettenkoferstrasse 11, Munich, 80336 Germany

## Abstract

That the human brain contains magnetite is well established; however, its spatial distribution in the brain has remained unknown. We present room temperature, remanent magnetization measurements on 822 specimens from seven dissected whole human brains in order to systematically map concentrations of magnetic remanence carriers. Median saturation remanent magnetizations from the cerebellum were approximately twice as high as those from the cerebral cortex in all seven cases (statistically significantly distinct, p = 0.016). Brain stems were over two times higher in magnetization on average than the cerebral cortex. The ventral (lowermost) horizontal layer of the cerebral cortex was consistently more magnetic than the average cerebral cortex in each of the seven studied cases. Although exceptions existed, the reproducible magnetization patterns lead us to conclude that magnetite is preferentially partitioned in the human brain, specifically in the cerebellum and brain stem.

## Introduction

Several studies documented the presence of magnetite (Fe_3_O_4_) in the human brain by its crystal habit^1–3^, X-ray or electron diffraction pattern^1,2^, the existence of a Verwey transition^4^ and isothermal remanent magnetization acquisition curves that saturate by 300 mT^1,4–7^. Magnetite grains were measured to grade in size between 1 to 70 nm, occasionally up to 200 nm, thus in the superparamagnetic to single domain range^1,3^. Some authors proposed that magnetite is a product of internal biomineralization and its ubiquitous presence serves a physiological function^8^. Others proposed that magnetite in the brain can originate from external sources, being absorbed through the nasal sinus cavity and olfactory bulb^3^. The latter study reported the existence of magnetite particles within the frontal cortex of 37 human post-mortem brains, with the implication that the presence of superparamagnetic magnetite is an environmental phenomenon derived from airborne particulate matter and not genetically-related. Although some work has focused on specific brain regions like the hippocampus^5,9^, none has carried out systematic mapping of magnetite of the human brain. Here, we present magnetization results from seven whole brains, three female and four male, aged from 54 to 87 years (Table 1).

**Table 1:** Statistical parameters for each human brain used in the present study.

Brain #	1 (54, m)		2 (56, m)		3 (68, m)		4 (73, m)		5 (87, f)		6 (79, f)		7 (84, f)	
SIRM data	cut off	all	cut off	all	cut off	all	cut off	all	cut off	all	cut off	all	cut off	all
cb med	30.4	18.8	66.3	75.3	62.1	349.1	31.0	30.5	21.2	27.9	30.0	33.5	37.7	57.4
cb μ*	34.6	11.9	54.7	67.5	97.5	231.2	29.6	28.2	27.1	34.1	30.6	32.9	31.3	61.4
cb σ*	3.1	5.5	1.9	2.2	3.0	3.2	1.6	1.7	1.8	2.0	1.6	1.6	1.9	4.0
cb N	14	22	13	18	4	12	12	16	8	16	12	16	10	15
cb wt (g)		140		88		152		107		84		97		78
cc med	14.4	12.1	18.8	19.2	37.6	56.2	10.4	8.4	13.9	16.5	17.4	16.7	19.8	20.7
cc μ*	16.7	12.4	20.3	23.1	37.2	59.7	10.9	9.3	12.8	16.0	20.6	17.0	20.1	23.3
cc σ*	2.7	2.9	3.2	3.8	2.6	3.6	2.4	3.4	1.8	2.0	2.5	2.7	2.7	3.6
cc N	76	97	59	93	65	109	66	103	34	81	68	95	69	88
cc wt (g)		863		752		1020		1080		699		714		636
cb μ*/cc μ*	2.07	0.96	2.69	2.93	2.62	3.87	2.73	3.04	2.11	2.13	1.49	1.94	1.56	2.64
p-value (MW)	**<0.001**	0.39	**<0.001**	**<0.001**	**0.05**	**<0.001**	**<0.001**	**<0.001**	**0.007**	**<0.001**	**0.03**	**0.001**	**0.05**	**0.004**

Abbreviations are: m/f, male/female; cb, cerebellum; cc, cerebral cortex; wt, total weight of measured pieces; μ*, geometric mean of the saturation isothermal remanent magnetization (SIRM) values in nAm^2^/kg; σ*, multiplicative standard deviation of SIRM in nAm^2^/kg; N, number of specimens used in calculation; med, median SIRM value in nAm^2^/kg; p-values given for the one-tailed Mann-Whitney (MW) test–values in bold are significant at the 0.05 level or better.

## Methods

The human post mortem brains investigated in the present study were collected between 1991 and 1995 in Heidelberg, Wiesloch and Bayreuth (Germany). They were donated by relatives and/or legal guardians of the decedents to one of us (H.H.) for examination and scientific research without time limit, in accordance with relevant guidelines and regulations in Germany. The use of these brains for scientific research was approved by the Ethical Board of the Faculty of Medicine at the University of Würzburg (Germany) in 1995. Little information was known about the subjects other than sex, birth and death dates; one of the male subjects was documented with schizophrenia. The removal of the brain specimens from the skull followed standard procedures of opening the cranial cavity with a saw and then cutting the dura matter and cervical medulla with a stainless steel scalpel. The brains were immersion fixed in 10% formaldehyde that was renewed every several years up until our investigation.

Each brain was dissected into roughly 8 cm^3^ cubes using a zirconia knife in a clean environment. Once removed from the formaldehyde bath, the arachnoid mater and the blood vessels that envelop the brain were removed, and then the brain was dissected using a combination of cuts along the horizontal, frontal and sagittal planes. First, the cerebellum plus brain stem were separated from the other parts of the brain at the level of the mesencephalon, and then the hemispheres were separated (left, L and right, R). Both hemispheres were cut into four horizontal levels from dorsal (I, top) to ventral (IV, bottom); each level was cut into three sagittal sections (for each hemisphere, 1 toward the midline and 3 toward the temporal poles); and finally cuts were made in rows from rostral (a, front) to occipital (e-g, back). The cerebellum was cut into two hemispheres (L and R) and each half into two horizontal levels (I and II), two sagittal cuts (1 and 2 [3 for brain #1]) and two rows (a and b). In this way, each specimen’s location was specified by a unique identifier in three dimensions based on its hemisphere, dorsal-ventral, medial-temporal and frontal-occipital position. This procedure yielded a total of 666 specimens from the cerebral cortex and 115 from the cerebellum for an average of 95 cerebral cortex and 16 cerebellum specimens per brain with an average weight of ~8 gm/specimen from those regions (Table 1). A few specimens separated into several small pieces after cutting and had to be discarded.

The brain stems from five brains [#3 to #7] were also included in our study. They were divided into pons and medulla oblongata; in four of the five [#4 to #7], the pons and medulla oblongata were further divided into the R and L halves (N = 18). We measured one olfactory bulb (herein bulb refers to bulb plus olfactory tract) for two brains [#1 and #2] and both the right and left olfactory bulbs in the other five [#3 to #7] (N = 12). The pineal gland from all seven brains was also analyzed (N = 7), as were the left and right choroid plexus (upper three were grouped into one) for two brains [#1 and #3] (N = 4).

Once cut, each specimen was placed into a sterile vessel, weighed, and transported to a magnetically shielded room (~200 nT ≈ < 0.5% of the Earth’s ambient field) situated in a forest, 80 km northeast of Munich, that houses a three-axis, superconducting magnetometer (bore diameter = 7.6 cm) (2G Enterprises Inc., Mountain View, CA, USA). This laboratory has the advantage of avoiding anthropogenic magnetic pollution pervasive in urban environments^10^. After the first three brains were measured, the shielded room was converted to a clean room complete with a high efficiency particulate air (HEPA) filter and electromagnetic filtered air; no systematic difference in magnetization characteristics (average intensity, etc.) was observed before or after conversion of the lab.

Three researchers participated in the magnetic measurements: one handled the containers, another transferred the specimens from the containers to a quartz glass sample holder and a third operated the magnetometer. In this way, all specimens came into contact solely by the same single, sterile tweezers by a researcher who handled only the tweezers. The same quartz glass holder was used for all specimens and was regularly monitored that its magnetization was negligible. The remanent magnetization of each specimen was first measured in its initial (natural) state (called NRM for natural remanent magnetization) and then returned to its vessel. After the NRM values for all pieces were measured, the specimens in their vessels were placed one at a time in an electromagnet and exposed to a 0.8 T field for a few seconds. All specimens were returned to the shielded room and their magnetic remanences were measured again in their magnetically saturated state (called SIRM, for saturation isothermal remanent magnetization); 10 to 300 minutes elapsed between SIRM acquisition and remanence measurement.

NRM and SIRM measurements were done by inserting the specimen into the magnetometer where its magnetic moment was measured twice in two different orientations before and after rotating 90° about a vertical axis in the magnetometer. The baseline of each component was subtracted from the measurements, the moments of each component (x, y and z) were averaged, and then a magnetic vector was calculated from the three components. After multiple measurements over several days and times, we found that we could reproduce two separate vector measurements when the magnetic moment was equal or higher than 3.75 × 10^−11^ Am^2^, although the level could be as low as 1.5-2.0 × 10^−11^ Am^2^ depending on the day or time of day. Thus, as a very conservative estimate, we consider any magnetic moment lower than 3.75 × 10^−11^ Am^2^ as below detection limits.

### Data availability

Data generated in this study can be obtained from the corresponding author in .xls or .pdf format.

## Results

**Figure 1:**
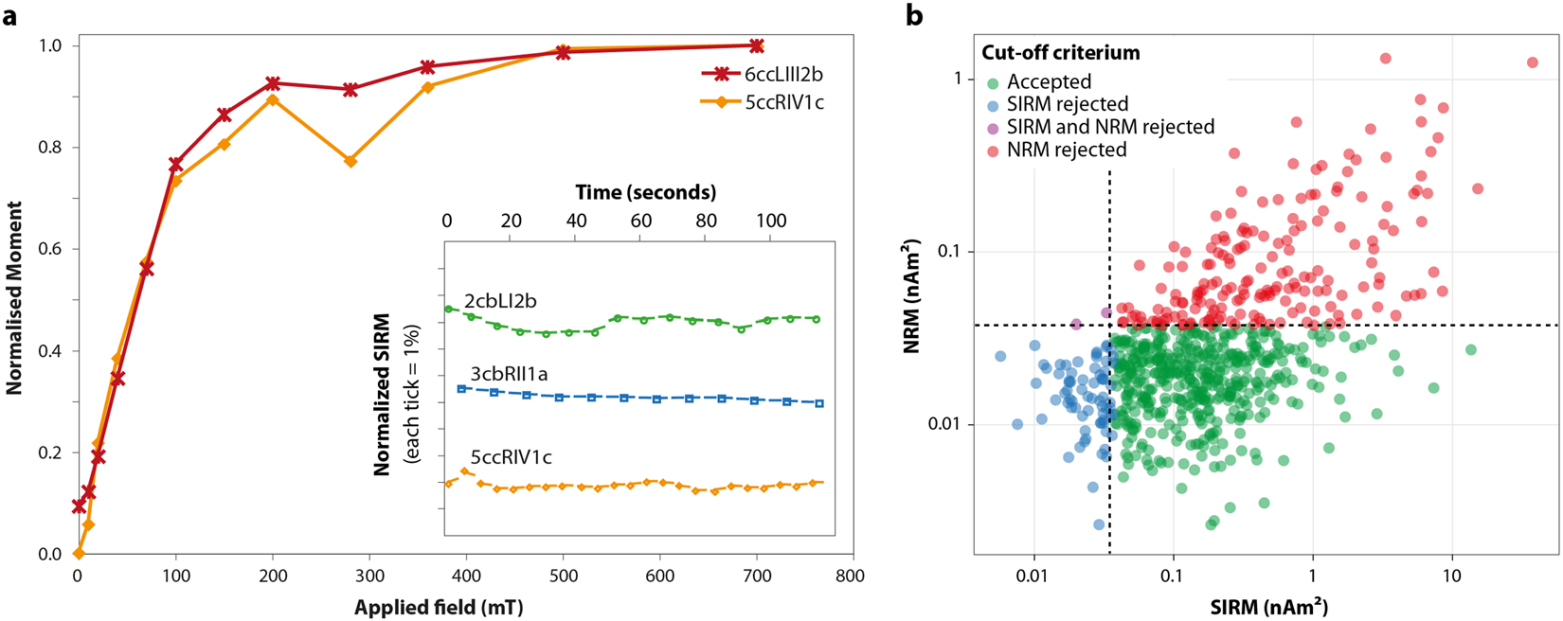
(**a**) Acquisition of saturation isothermal remanent magnetization (SIRM) for two cerebral cortex (cc) specimens. The insert shows the change in magnetization as a function of time for two cerebellum (cb) specimens and one cerebral cortex specimen after being exposed to a 0.8 T field. Sample identification begins with brain number, cc or cb, hemisphere (L or R), horizontal level (I–IV), sagittal section (1–2) and row (a–c). (**b**) Log-log plot of natural remanent magnetization (NRM) versus SIRM for all measured samples (non-mass normalized). Dashed lines indicate levels of 3.75 × 10^−11^ Am^2^. The cut-off method accepted the data points in green; red and blue points were rejected based on the NRM or SIRM cut-off criteria as described in the text.

We first carried out stepwise isothermal remanent magnetization acquisition experiments (Fig. 1). All measured samples were essentially saturated by 300 mT, consistent with the presence of magnetite as previously found^1,2,4,5^. The magnetizations of a few samples were measured over time beginning one minute after exposure to a 0.8 T field at room temperature to test for magnetic viscosity (Fig. 1), which decays logarithmically with time^11^ depending on grain size, temperature, applied magnetic field intensity and time of exposure to the applied field^12^. The absence of magnetic viscosity demonstrated that the SIRM values reported in the present study were not biased by the relative timing between application of the 0.8 T field and measurement of the magnetic moment; e.g., not influenced by superparamagnetic grains.

Some specimens had macroscopically visible blood vessels within them. We investigated whether the blood vessels held the magnetic remanence by measuring the magnetizations of the brain specimens with the blood vessels and then again after removing them. No measureable remanence was found in the vessels; i.e., the magnetic remanence of the brain specimen remained the same after removing the blood vessels and hence, the magnetite resided within the brain tissue.

Mass normalized SIRM values were analyzed based on two different data selection criteria (Tables 1 and 2; Fig. 1b): (1) accepting all measured specimens and (2) accepting only specimens that carried initially no magnetic remanence (thus had NRM moments below the detection limit of 3.75 × 10^−11^ Am^2^) and that acquired a measurable magnetic remanence with SIRM moments above the detection limit. Rejecting specimens with NRMs above the detection limit mitigated potential contamination effects (e.g., metal fragments shed from the saw that cut through the skull) by assuming that the pristine human brain will not carry a natural remanence, whereas magnetized particles from contamination likely possess detectable NRM moments. Rejecting specimens with SIRM moments below the detection limit reduced a bias that may arise through mass normalization, as dividing small SIRM moments by very small masses can lead to large apparent magnetizations. Applying these NRM and SIRM cut-off criteria (below referred to as the cut-off method) represents the most conservative way to examine the data.

**Table 2:** Number and type of outliers for the cerebral cortex and cerebellum defined by the cut-off method.

Brain #	N, NRM outliers	% of total	Median NRM (Am^2^)	N, SIRM outliers
1	—	—	—	29
2	31	26	5.66 × 10^−11^	9
3	48	40	8.10 × 10^−11^	4
4	14	12	6.05 × 10^−11^	27
5	54	55	5.73 × 10^−11^	3
6	16	14	5.24 × 10^−11^	15
7	15	14	10.16 × 10^−11^	9
TOTAL	178	26	—	96
L	96	28	6.18 × 10^−11^	56
R	82	25	6.17 × 10^−11^	40
cc only				
I	10	11	5.93 × 10^−11^	21
II	30	19	5.87 × 10^−11^	32
III	48	27	6.21 × 10^−11^	23
IV	59	39	7.09 × 10^−11^	9
1	74	31	6.07 × 10^−11^	20
2	53	23	8.36 × 10^−11^	47
3	20	18	6.13 × 10^−11^	18
a	29	35	9.60 × 10^−11^	14
b	27	23	6.58 × 10^−11^	15
c	31	24	5.70 × 10^−11^	17
d	25	20	7.09 × 10^−11^	20
e	23	25	5.92 × 10^−11^	10
f	12	32	9.63 × 10^−11^	9

Abbreviations are: N, number; NRM, natural remanent magnetization; SIRM, saturation isothermal remanent magnetization; cc, cerebral cortex; cb, cerebellum; L, left hemisphere; R, right hemisphere; % of total, number of NRM outliers divided by the total number of measured specimens in a given location (in percent); I–IV, horizontal level; 1–3, sagittal plane; a–f, row. Note that brain #1 had no NRM values so outliers were only based on the SIRM criterion for the cut-off method.

The second (cut-off) method serves an important verification tool, so we explain it here in detail using two examples. Specimen 5ccRIII2a came from the rostral-most (a, frontal) position on the third horizontal level from the middle (2nd) section of the right side (R) of the cerebral cortex (cc) of brain #5. This specimen had an obvious saw mark. Its non-mass normalized NRM moment was 5.66 × 10^−10^ Am^2^, well above the detection threshold of the magnetometer and over an order of magnitude higher than the median non-mass normalized NRM moment of the other specimens from same level (III) on the right side of the cerebral cortex of the same brain (3.80 × 10^−11^ Am^2^, N = 13). The non-mass normalized SIRM moment of 5ccRIII2a was 7.59 × 10^−10^ Am^2^, similar to the NRM value, meaning that the specimen was essentially at saturation before application of a 0.8 T field. The first method (all data) accepted the mass normalized SIRM value from specimen 5ccRIII2a (1.21 × 10^−07^ Am^2^/kg, based on a mass of 6.2993 g) while the second (cut-off) method rejected it because the first requirement, that the non-mass normalized NRM must be <3.75 × 10^−11^ Am^2^, was not fulfilled.

The second example comes from the right olfactory bulb of brain #6 (6olR). Its non-mass normalized NRM moment was 1.53 × 10^−11^ Am^2^, thus passing the first condition of the cut-off method. Its non-mass normalized SIRM moment was 1.65 × 10^−11^ Am^2^, but because of its low mass (0.1302 g), its mass normalized SIRM moment appeared very high, 1.27 × 10^−07^ Am^2^/kg, even higher than 5ccRIII2a. Specimen 6olR was rejected by the cut-off method based on the second requirement—that the non-mass normalized SIRM moment should be > 3.75 × 10^−11^ Am^2^—was not fulfilled. If the magnetometer drift was low at the time of measurement, then 1.65 × 10^−11^ Am^2^ could be a significant measurement and the mass normalized value would have physical meaning. On the other hand, if the drift was above 1.65 × 10^−11^ Am^2^, then the SIRM moment had no physical meaning, merely being an artifact stemming from the relatively low mass of the sample and the noise level of the magnetometer.

Considering the 666, 115 and 18 measured specimens for the cerebral cortex, cerebellum and brain stem, respectively, the mass normalized SIRM values ranged from 9.8 × 10^−10^ to 3.0 × 10^−6^ Am²/kg with a median of 2.04 × 10^−8^ Am²/kg. Applying the cut-off method rejected 35% of the measured specimens and yielded N = 437, 73 and 11 for the cerebral cortex, cerebellum and brain stem, respectively, with mass normalized SIRM values ranging from 2.4 × 10^−9^ to 1.8 × 10^−6^ Am²/kg and a median of 1.97 × 10^−8^ Am²/kg (Table 1). Assuming the median SIRM of ~2 × 10^−8^ Am^2^/kg was derived from randomly-oriented single domain magnetite suggests the brain contains ~10^−9^ kg of single domain magnetite per kilogram of brain tissue (one ppb); if individual crystals have volumes of 50^3^ nm^3^, then ~10^9^ single domain grains per kilogram reside in the brain tissue. In most cases, median values of both datasets were similar for all seven brains combined, as well as for each individual brain sample, but the standard errors were reduced when applying the cut-off method (Table 1). Of the two conditions used for the cut-off method, the NRM exclusion (NRM moment above threshold) was invoked nearly twice as often as the SIRM exclusion (SIRM moment below threshold) (Table 2). Due to the relatively large specimen masses used in our study, SIRM moments were most often above the magnetometer sensitivity. Figure 1b plots NRM versus SIRM for all specimens and indicates which of those specimens were omitted using the cut-off method.

**Figure 2:**
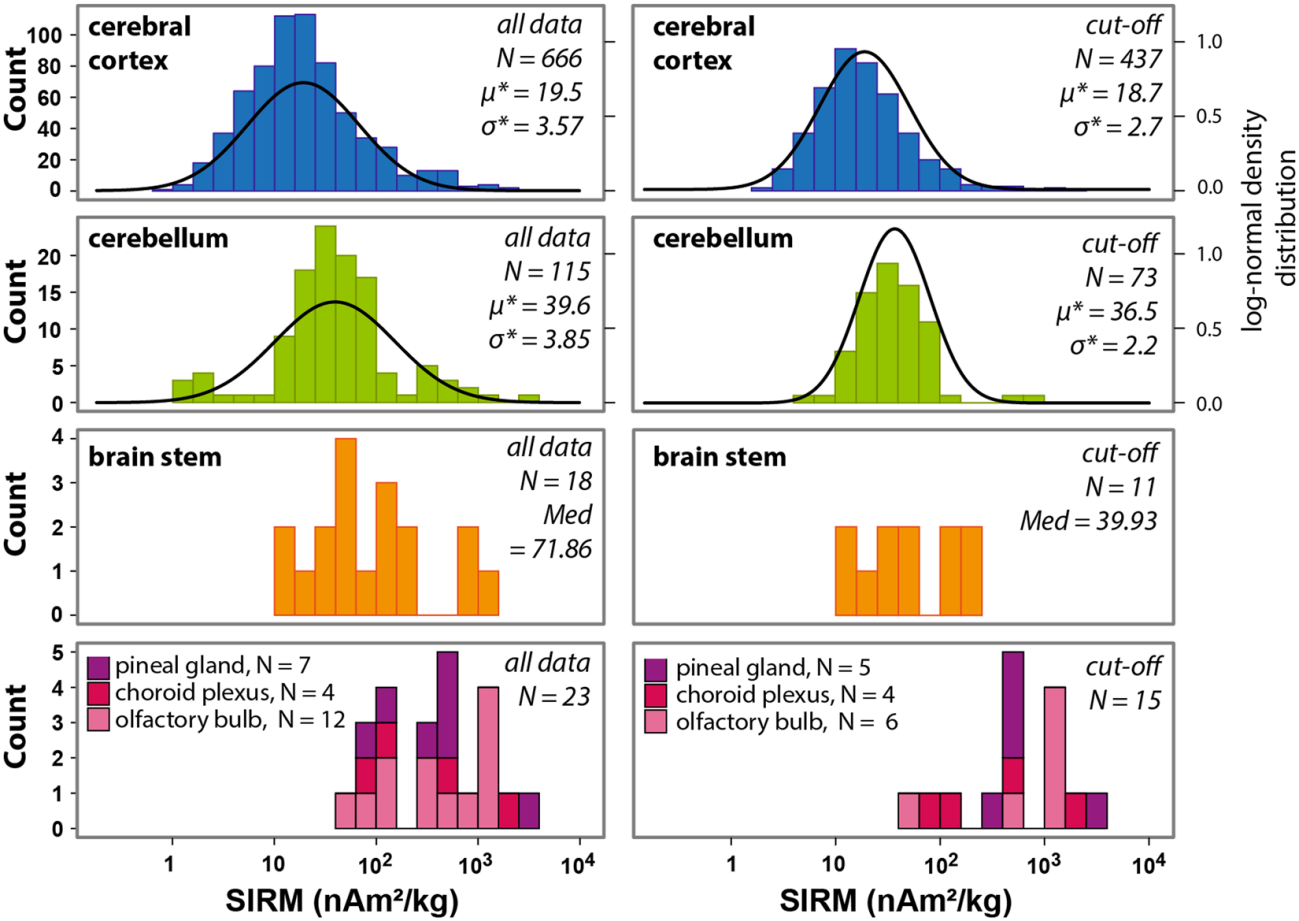
Histograms of the mass normalized saturation isothermal magnetizations (SIRM) separating the specimens by anatomy. Data are shown using all values (histograms on the left) and after applying the cut-off method (histograms on the right). Log-normal density distribution fits to the data are provided for the cerebral cortex and cerebellum. Abbreviations are: N, number of specimens; μ*, geometric mean in nAm^2^/kg; σ*, multiplicative standard deviation in nAm^2^/kg; Med, median in nAm^2^/kg.

Figure 2 shows the SIRM distributions of all specimens from the seven brains classified according to their anatomical positions. Although SIRM values among the specimens of each brain spanned roughly three orders of magnitude, subdividing the dataset into the different anatomical regions revealed a fairly systematic pattern of magnetization intensities. Histograms indicate that the SIRM data were approximately log-normally distributed for the cerebral cortex and cerebellum that had relatively high numbers of specimens (Fig. 2; Table 1). Median cerebellum SIRMs were 3.12 ± 1.65 higher than the SIRMs of the cerebral cortex taking all data and 2.20 ± 0.76 higher considering the cut-off values (Table 1). Median SIRM values of the brain stem samples, taken together due to their relatively low numbers, were 4.23 (all data) and 2.38 (cut-off data) higher than those for the cerebral cortex (Fig. 2). The median SIRM magnetizations for the olfactory bulb, pineal gland and choroid plexus were over an order of magnitude higher than those for the cerebral cortex (Fig. 2).

**Figure 3:**
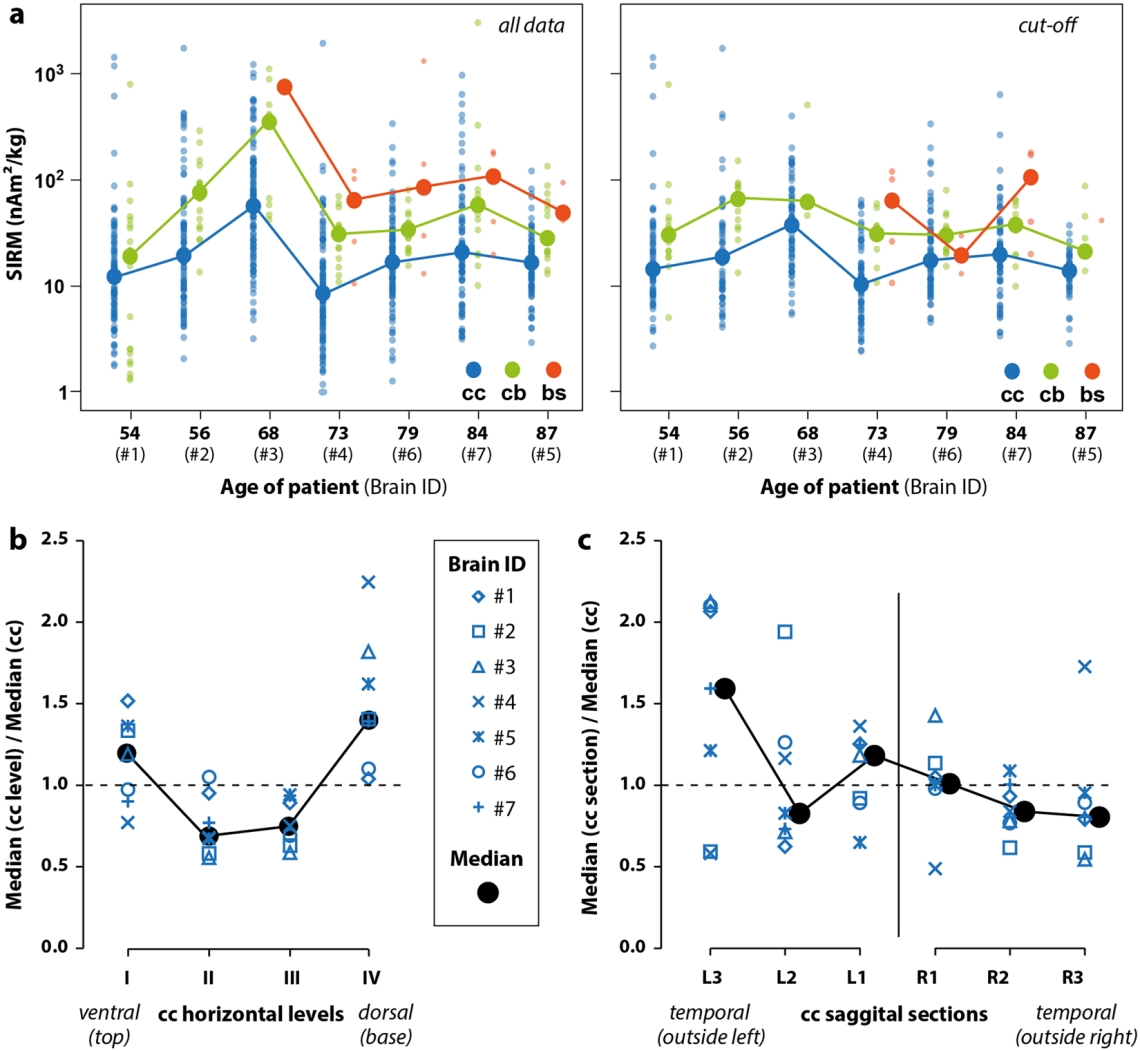
(**a**) Median, mass normalized, saturation isothermal remanent magnetization (SIRM) values of the cerebral cortex (cc), cerebellum (cb) and brain stem (bs) for each of the seven brains classified according to the person’s age using all data (left) and after using the cut-off method (right). Individual data points are small circles; medians are large circles. (**b**) Median SIRM of each level (I-IV) in the cerebral cortex divided by the median SIRM of the entire cerebral cortex for each individual brain using the cut-off method. Horizontal level IV (ventral, basal) in each brain was systematically more magnetic than the median value and levels II and III were less magnetic. (**c**) Median SIRM of each sagittal section of the cerebral cortex segregated by hemisphere (left, L and right, R) divided by the median SIRM of the entire cerebral cortex for each individual brain (cut-off method). The left hemisphere had higher magnetization in general and more variability, with the temporal (outside, L3) area generally being more magnetic whereas the temporal region of the right side (R3) was less magnetic. Median magnetizations between the left and right hemispheres were statistically significantly different from each other (p = 0.047).

Figure 3a ranks the data by individual brain as a function of age. Although the sample population was relatively small, the median magnetization of each of the seven brains appeared similar regardless of age or sex, except for brain #3, which was two to four times more magnetic than the rest (cut-off). Of note is that brain #3 came from a patient diagnosed with schizophrenia.

That all seven brains systematically concentrated magnetite in specific places was the most important outcome of our work (Fig. 3). In all individuals, the cerebellum was roughly twice as magnetic as the cerebral cortex. A Wilcoxon signed-rank test confirmed that this difference was statistically significant (p = 0.016) using all or cut-off data. A one-tailed Mann-Whitney test on the individual SIRM values of the cerebellum and cerebral cortex for each individual brain confirms that the two were statistically different (p values in Table 1), except for the case treating all specimens from brain #1. The median magnetization of the brain stem was always higher than the median magnetization of the cerebellum when the number of brain stem specimens exceeded two (Fig. 3a). Low numbers of brain stem specimens prohibited a meaningful statistical test on individual brains.

Looking at the cerebral cortex in detail, we calculated the median magnetization of each horizontal, frontal and sagittal plane for each individual brain, and then divided those values by the median magnetization for the whole cerebral cortex of the same brain. This showed that the ventral (basal, IV) level of the cerebral cortex was consistently more magnetic than the median cerebral cortex taken as a whole in all seven brains, while levels II and III were lower than average in 6 of 7 and 7 of 7 cases, respectively (Fig. 3b). Wilcoxon signed-rank tests comparing the median cerebral cortex against the medians of the different levels confirmed the systematic difference in magnetizations for levels II, III and IV (p = 0.016, 0.046 and 0.016, respectively). The left hemisphere of the cerebral cortex was more magnetic than the right in six of seven cases (Fig. 3c). A Wilcoxon signed-rank test indicated that the difference in the median SIRMs between the left and right hemispheres was statistically significant (p = 0.047).

**Figure 4:**
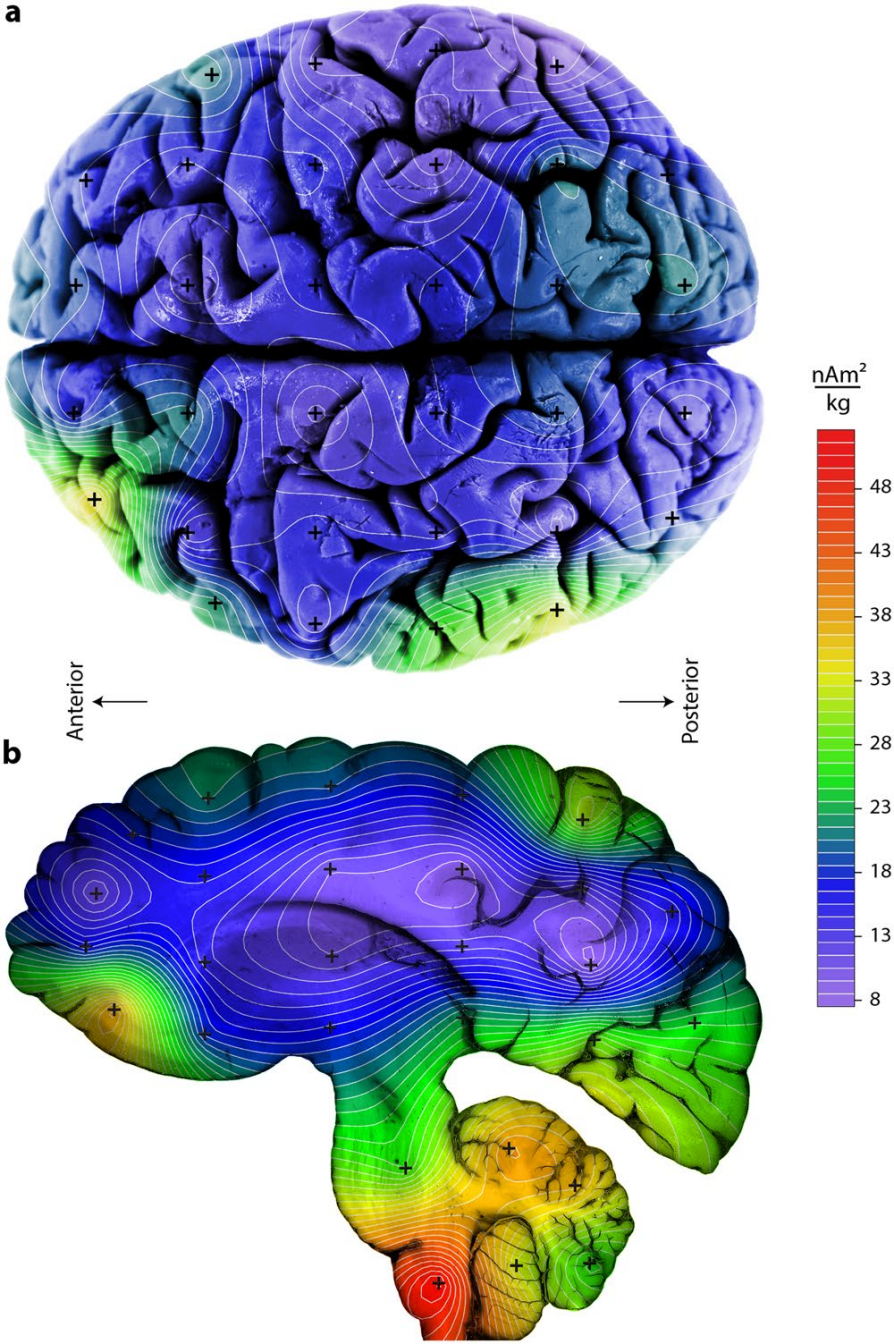
Contour maps using the Ferret (http://www.ferret.noaa.gov/Ferret) color scale of the median, mass normalized, saturation isothermal remanent magnetization (SIRM) values using the cut-off method. (**a**) Horizontal view from dorsal (above) of the cerebral cortex only. (**b**) Mid-sagittal view of the entire brain including cerebral cortex, cerebellum and brain stem. All pieces for all seven brains in a given location (plus symbols) were stacked along the projection to calculate the median at each location. Medians at each place were retained only when N ≥ 3. The linear color scale is the same for both images, which were made with Adobe Photoshop CS6 v 13 by overlaying the contour images generated by our own python code on top of our own photo (**a**) and scanned image (**b**).

Figure 4 summarizes our findings by calculating the median magnetization (cut-off method) of all specimens in a given anatomical location according to the projection and then contouring the values. Considering only the cerebral cortex observed from above, one sees that the magnetization was highest on the temporal (outside) left hemisphere and that the occipital (back) part of the cerebral cortex tended to be more magnetic than the rostral (front) region. The sagittal projection in Fig. 4 indicates that the brain stem and cerebellum had higher magnetization intensities than the cerebral cortex.

## Discussion

The present study was performed on human post mortem brains and has therefore inherent limitations. For example, cutting through the skull to extract the brains could have introduced magnetic contaminants. Some peripheral cerebral cortex specimens had visible saw marks a few mm deep that had NRMs well above detection limits; however, other specimens with cut marks had NRMs below detection limits. Samples with NRMs above detection limits would be omitted when considering cut-off data. Our findings that visible blood vessels did not hold the magnetic remanence corroborated previous results that cauterization of blood vessels did not increase the magnetic fraction^5^.

Only formalin fixed tissue was used in our study, which has important disadvantages. Storage in formalin has been found to categorically reduce total iron concentration in tissue as well as decrease remanent magnetization^5,13,14^. However, the extent of Fe removal and the source of the Fe that was removed are unclear. For example, Fe concentrations in eight brain specimens stored in formalin for an average of four years and then immersed in concentrated nitric acid for ~12 hours were 40% lower than non-formalin-fixed brain specimens^14^. A study of human spleen tissue bathed in formalin for 200 days found that 3% of the iron leached out of the tissue over the first 60 days thereafter the iron concentration stayed constant^13^. Mössbauer spectra before and after the 200-day immersion in formalin showed no evidence for chemical transformation of the remaining iron^13^. The remanent magnetization of brain tissue stored for a week in formalin became reduced, although its coercivity spectrum remained constant^5^. In our study, the absolute magnetite concentrations are most likely underestimated. However, the brains we used were stored in formalin over similar time periods, having been extracted between 1991 and 1995, and have similar median magnetizations (except brain #3), so it seems reasonable to compare magnetizations between samples stored over comparable times^7^. Moreover, formalin storage should not impact the relative distribution of the magnetization in individual brains stored as whole organs, nor contribute to asymmetrical distributions in the same anatomical structure (i.e., cerebral cortex; Fig. 4), so we conclude that the influence of formalin on the relative distribution of the remanence carriers was negligible.

Mass normalized SIRM values measured in our study were equal or less than previously reported SIRM values measured at room temperature^1,5^, and our sample masses were about eight times greater, which implies that errors due to uncertainties from mass normalization were mitigated in our study. The total mass of material used in our study exceeded most previous studies by two orders of magnitude. While laboratory tools and sample containers can contain magnetic contaminants^15^, it is difficult to imagine why those contaminants would systematically correlate with brain anatomy in seven different human beings. It is critical to note that asymmetric and systematic distribution of the SIRM magnetization discounts effects from long-term exposure in formaldehyde, chemical reactions from medication, preparation, etc.

The systematically distinct and statistically significant placement of the magnetic carriers in all seven brains could argue that they were biologically-engineered. Although the genetic expression for magnetite in the human brain is unknown, the mamL gene was found in all magnetite producing magnetotactic bacteria and likely plays an important role in magnetite biomineralization^16^. The magA gene in magnetotactic bacteria involved with iron transport is expressed in the human cell line 293FT, which aids in the production of magnetic, iron oxide nanoparticles by those cells^17^.

On the other hand, one study provided evidence that magnetite within the frontal lobe enters the central nervous system through the olfactory bulb^3^. Our measurements of the olfactory bulb showed they were over an order of magnitude more magnetic on average than the cerebral cortex (Fig. 2). However, the pineal gland and choroid plexus that are topographically situated far from the olfactory bulb and have no direct connection to it, were just as magnetic, so higher magnetization in itself does not seem diagnostic of magnetite absorbed from the environment (Fig. 2). If magnetite entered the body through the olfactory system, one would expect higher magnetite concentrations to exist in the frontal cortex closest to the olfactory bulb with a gradient to lower concentrations away from it. Such a diffusion pattern was absent in the SIRM distributions of all seven brains (Fig. 4). Although many of the specimens above the NRM threshold came from horizontal, frontal and sagittal positions around the olfactory bulb (basal, central, and rostal-most position IV, 1, a) (Table 2), this pattern only emerged when summing over all investigated brains, as individual brains showed no such trend.

For magnetite introduced from the environment, one would expect magnetite concentrations in the body to increase with the life span of the individual. This is generally consistent for male subjects, but not female subjects, in one study^8^. Frontal cortices of persons 3 to 85 years of age who lived in Mexico City exhibited no age-related trend^3^. In our study, linear regression of the median magnetizations of the cerebral cortex and cerebellum samples combined for each brain versus age of the person yielded an essentially flat, albeit slightly negative, slope based on either all data or cut-off values. That our data exhibited no statistically significant age-related correlation for persons aged between 54 and 87 years is compatible with chemical analyzes of non-haemin iron in the human brain^18,19^. In a study of 81 persons, iron concentrations increased sharply until 25–35 years of age followed by a gentle or null increase in iron thereafter^18^. The number of NRM outlier specimens in our study exhibited no statistically significant positive correlation with age (R² = 0.0003; Table 2); in fact, the trend was slightly negative (−0.026).

If the NRM values above the baseline were not related to contamination when the skull was cut with the saw to extract the brains, by absorbing magnetite through the olfactory bulb, etc., then the question arises why human brain tissue would contain magnetic remanence carriers whose individual moments were statistically aligned to produce a net magnetization. We recall that magnetotactic bacteria grow identical magnetite crystalline morphotypes as found in the human brain^1^. Not only do magnetotactic bacteria make among nature’s best single domain magnetite, they genetically engineer the magnetite to grow in chains with the magnetic easy axes of the individual magnetosomes aligned parallel to one another. This construction leads to a permanent net magnetization that enables magnetotactic bacteria to swim along magnetic field lines^20^. A similar construction could arise in humans. Lobsters^21^ and pigeons^22^ were also found to have significant NRMs. Another remotely plausible explanation for measurable NRM values is that the head of the living person or the extracted brain during storage or transport was exposed to a strong magnetic field such that the region in the brain close to the external field became magnetized in a manner analogous to a SIRM. However, this interpretation has difficulty explaining why some isolated NRM outlier specimens existed well within the interior region of the cerebral cortex (Table 2).

All seven human brains in our study yielded systematic enrichment in permanent magnetic remanence carriers toward the ventral and caudal regions, which seems to support a predominantly internal origin for the remanence carrying material. If the magnetite has a biological origin, then finding which cells contain the magnetite becomes of paramount importance. While birds are thought to have integrated magnetic sensors to aid orientation^23,24^, for humans, magnetite’s purpose is unclear. This has to be established in future studies.
